# cGAS: bridging immunity and metabolic regulation

**DOI:** 10.1093/jmcb/mjaf018

**Published:** 2025-07-17

**Authors:** Jing Wang, Wen Meng

**Affiliations:** National Clinical Research Center for Metabolic Diseases, The Second Xiangya Hospital of Central South University, Changsha 410011, China; The Metabolic Syndrome Research Center, The Second Xiangya Hospital of Central South University, Changsha 410011, China; Department of Metabolism and Endocrinology, The Second Xiangya Hospital of Central South University, Changsha 410011, China; National Clinical Research Center for Metabolic Diseases, The Second Xiangya Hospital of Central South University, Changsha 410011, China; The Metabolic Syndrome Research Center, The Second Xiangya Hospital of Central South University, Changsha 410011, China; Departments of Oncology, The Second Xiangya Hospital of Central South University, Changsha 410011, China

## Abstract

Recent advances have revealed that cyclic guanosine monophosphate–adenosine monophosphate (AMP) synthase (cGAS), classically recognized as a cytosolic DNA sensor, plays crucial roles beyond innate immunity. Particularly in the adipose tissue, cGAS functions as a metabolic sentinel, responding to mitochondrial stress and contributing to inflammation, insulin resistance, and energy imbalance. These effects occur through both stimulator of interferon genes (STING)-dependent and STING-independent pathways, involving autophagy, chromatin remodeling, and transcriptional reprogramming. Here, we propose a paradigm shift positioning cGAS at the intersection of immunity and metabolism. We explore its multifaceted roles in adipocytes and other metabolic tissues, highlighting emerging therapeutic opportunities and future research directions.

## Introduction

Cyclic guanosine monophosphate–adenosine monophosphate (AMP) synthase (cGAS) is best known as a cytosolic DNA sensor that initiates stimulator of interferon genes (STING)-dependent type I interferon responses to the presence of double-stranded DNA in the cytoplasm, typically derived from pathogens or damaged host cells (Chen et al., 2016; Bai and Liu, 2021). However, homologs of cGAS exist in bacteria and invertebrates, implicating primordial roles in cellular homeostasis beyond immunity (Jenson et al., 2023; Jenson and Chen, 2024). Indeed, evolutionary insights and recent discoveries suggest broader functions of the components in this signaling pathway. In adipocytes, mitochondrial DNA (mtDNA) release under metabolic stress activates cGAS, linking it to inflammation, thermogenic suppression, and insulin resistance (Bai et al., 2020; Gong et al., 2024). Activation of this pathway has also been implicated in metabolic dysfunction in other tissues (Maekawa et al., 2019; Donne et al., 2022; Li et al., 2023; Ma et al., 2024; Wang et al., 2024a; Jiao et al., 2025). These findings suggest that cGAS acts as a metabolic regulator, regulating energy homeostasis beyond its canonical immune role.

This dual functionality of cGAS reflects a broader principle in disease pathogenesis: the tight coupling between innate immunity and metabolism. Chronic low-grade inflammation, often initiated by innate immune sensors, contributes to obesity, insulin resistance, and fatty liver disease. Conversely, metabolic stress—such as nutrient excess or mitochondrial dysfunction—can activate immune pathways like cGAS–STING, establishing a bidirectional immunometabolic feedback loop (Gong et al., 2024; Liu and Liu, 2024).

## cGAS–STING signaling in metabolism

In response to mitochondrial stress, adipocytes release mtDNA into the cytosol, which activates cGAS and triggers a cascade of metabolic reprogramming events (Bai et al., 2017). Notably, cGAS activation suppresses the expression of thermogenic genes such as *UCP1* and *PGC1α* by elevating cyclic nucleotide phosphodiesterase (PDE) activity, leading to reduced intracellular cyclic AMP (cAMP) levels and inhibiting protein kinase A (PKA)-mediated signaling (Bai et al., 2020). This mechanism directly links cGAS to impaired energy expenditure and the progression of obesity.

Emerging evidence also suggests that cGAS activation in adipocytes exacerbates insulin resistance through two interrelated mechanisms: inflammatory priming and lipid overload. First, cGAS activation induces the secretion of pro-inflammatory cytokines, including interleukin-6 (IL-6) and tumor necrosis factor-α (TNF-α), which disrupt insulin receptor substrate phosphorylation and impairs downstream phosphatidylinositol 3-kinase (PI3K)/protein kinase B (AKT) signaling pathway, thereby promoting insulin resistance and amplified inflammation in the adipose tissue (Bai et al., 2017, 2020). Second, sustained cGAS activation enhances lipolysis and increases the release of free fatty acids, further compromising adipose tissue plasticity and systemic glucose homeostasis (Gong et al., 2024). Together, these findings highlight cGAS as a key integrator of mitochondrial dysfunction, inflammation, and metabolic dysregulation. Through both inflammatory and metabolic pathways, cGAS disrupts adipose tissue homeostasis and contributes to systemic metabolic dysfunction.

## STING-independent functions of cGAS: new mechanistic insights

### Autophagy and mitochondrial integrity

While cGAS classically signals through STING to mount type I interferon responses, accumulating evidence reveals that cGAS also exhibits diverse STING-independent functions that directly influence cellular metabolism.

cGAS interacts directly with Beclin-1, displacing Rubicon and initiating autophagy through the class III PI3K complex, independent of STING (Liang et al., 2014). This pathway supports mitochondrial quality control, particularly under acute stress such as hepatic ischemia–reperfusion injury, and mitigates oxidative damage by mitophagy (Lei et al., 2018).

Beyond autophagy, cGAS also influences mitochondrial dynamics. Mitochondrial stress triggers mtDNA release and cGAS–STING activation, which in turn engages feedback loops that restrain excessive mitochondrial biogenesis—thus preventing organelle overloading. This dynamic is underpinned by the mitophagy–biogenesis crosstalk essential for mitochondrial homeostasis (Liu et al., 2023) and is consistent with emerging models of mtDNA-driven immune sensing that regulate organelle quality control (VanPortfliet et al., 2024). In colorectal cancer, cGAS further suppresses oxidative phosphorylation (OXPHOS) while promoting glycolysis through its interaction with NDUFA4L2, a mitochondrial complex I subunit, thereby reprogramming tumor cell metabolism without STING involvement (Wang et al., 2024b). This indicates that cGAS may function as an energy ‘rheostat’, balancing energy production with the risk of organelle damage, and this process can occur independently of STING.

### Chromatin remodeling and transcriptional reprogramming

It has recently been shown that chromatin-bound cGAS (ccGAS) recruits the SWI/SNF complex to modulate genes involved in glutaminolysis and DNA replication (Lv et al., 2024). This epigenetic regulation functions independently of enzymatic activity or STING, underscoring cGAS's role in cellular adaptation to metabolic stress.

### The hypoxia–cGAS axis in obesity and cancer

In obesity, adipose tissue expansion outpaces vascular growth, leading to local hypoxia and stabilization of hypoxia-inducible factor 1α (HIF-1α). Activated HIF-1α drives inflammation, fibrosis, and metabolic dysfunction by promoting pro-inflammatory cytokines, remodeling extracellular matrix, and impairing mitochondrial function. This hypoxia–HIF-1α axis plays a central role in linking adipose tissue dysfunction to systemic insulin resistance and obesity-related diseases. Notably, chronic low-grade inflammation and hypoxia in obese adipose tissue also create a pro-tumorigenic microenvironment, contributing to the development and progression of obesity-associated cancers such as colorectal, breast, and liver cancers (Divella et al., 2016; Brown and Scherer, 2023).

The interplay between cGAS and HIF isoforms highlights the emerging role of cGAS in metabolic regulation and cancer development. While HIF-2α reduces ceramide levels and protects against vascular disease, HIF-1α promotes ceramide synthesis and insulin resistance (Zhang et al., 2019b; Wang et al., 2022). In colorectal cancer, cGAS activity is also influenced by hypoxia and lactylation, linking cGAS to ceramide metabolism, vascular inflammation, and immune evasion (Li et al., 2024; Rao et al., 2025). These STING-independent functions position cGAS as a key regulator of cellular metabolism and stress responses. Dysregulation of these pathways contributes to adipose dysfunction, insulin resistance, and fatty liver disease, suggesting that targeting cGAS and HIF signaling could provide new therapeutic strategies for obesity-related conditions and cancer (Bai et al., 2017; Zhang et al., 2019b; Gong et al., 2024; Li et al., 2024).

## cGAS and the metabolic–immune trade-off

### Balancing autophagy and inflammation

The evolutionary conservation of cGAS-like sensors from bacteria to mammals suggests that their original function extends beyond immune defense, supporting the emerging concept of a ‘metabolic–immune trade-off’ proposed in systems biology frameworks (Liu and Liu, 2024). This paradigm posits that cells dynamically allocate resources between immune surveillance and metabolic homeostasis, with cGAS acting as a regulator in this balance, particularly in stressed adipocytes. Under moderate mitochondrial stress, cGAS binds to Beclin-1 to activate autophagy in a STING-independent manner, promoting the clearance of damaged mitochondria and preserving metabolic efficiency (Liang et al., 2014). However, under chronic stress, persistent mtDNA leakage exceeds this adaptive capacity and signaling shifts toward STING-dependent inflammation. Such a transition reflects a broader systemic trade-off, where a metabolic checkpoint is governed by stress magnitude and sustained metabolic stress drives a shift from maintenance to immune activation. In adipocytes, this transition contributes to insulin resistance, while in T cells, STING activation under low FADS2 activity leads to PUFA depletion and loss of suppressive function (Vila et al., 2022). Thus, cGAS–STING acts as a sensor-integrator of energetic stress and immune adaptation.

### Thermogenesis suppression

By activating PDE3B/4 and suppressing cAMP–PKA signaling, cGAS reduces thermogenesis in adipocytes (Bai et al., 2020). This mirrors immunometabolic prioritization observed in macrophages, where energy is redirected from metabolism to immune defense, exacerbating insulin resistance. In adipocytes, this shift exacerbates adiposity and insulin resistance, creating a positive feedback loop where metabolic stress further amplifies cGAS activation.

### cGAS functions in other metabolic tissues

cGAS has also been shown to play important roles in regulating the functions of other tissues and organs. cGAS in the liver is activated during metabolic dysfunction-associated fatty liver disease (MAFLD), likely in response to DNA damage from replication stress caused by nucleotide pool imbalance (Donne et al., 2022). Exercise decreases CHCHD4 levels to limit TRIAP1 import into mitochondria and increase mtDNA release, which activates cGAS–STING/NF-κB signaling and promotes oxidative fiber formation in the skeletal muscle (Ma et al., 2024). These findings suggest that cGAS may support muscle adaptation and offer therapeutic potential in metabolic conditions. In the kidney, cGAS activation contributes to both acute and chronic kidney injury by promoting inflammation and fibrosis (Maekawa et al., 2019; Li et al., 2023; Wang et al., 2024a; Jiao et al., 2025). cGAS signaling in the heart has been linked to lipid metabolism disorders and mitochondrial dysfunction in cardiometabolic diseases (Yan et al., 2022; Mao et al., 2023). In pancreatic islets, cGAS modulates CEBPβ expression independently of STING, promoting β-cell proliferation and supporting glucose regulation (Cai et al., 2024). These findings highlight the diverse roles of cGAS across metabolic tissues and underscore the need for further research to fully understand its metabolic functions.

### cGAS–STING signaling and cellular energy metabolism

The cGAS–STING pathway is increasingly recognized as a key regulator of cellular energy metabolism across multiple substrates. Activation of cGAS–STING suppresses OXPHOS and promotes aerobic glycolysis in cancer and inflamed tissues, supporting a metabolic shift akin to the Warburg effect (Field et al., 2020; Casadio, 2022). In colorectal cancer cells, cGAS directly interacts with NDUFA4L2 to inhibit mitochondrial complex I and reprogram metabolism independently of STING (Wang et al., 2024b).

Fatty acid metabolism is also modulated by cGAS–STING activation. In adipocytes, cGAS suppresses thermogenesis by reducing cAMP–PKA signaling, which inhibits fatty acid oxidation and promotes lipid accumulation (Bai et al., 2020). In the cardiac tissue, mitochondrial cholesterol dysregulation activates cGAS–STING and contributes to cardiometabolic diseases (Mao et al., 2023; Zhang et al., 2024). Cholesterol and ceramide metabolism are further implicated through HIF-regulated crosstalk with cGAS: HIF-1α promotes ceramide synthesis and inflammation, while HIF-2α enhances ceramide degradation and improves metabolic outcomes (Zhang et al., 2019b; Wang et al., 2022).

Glucose and amino acid metabolism are integrated into the cGAS regulatory network as well (Hu et al., 2022; Saha et al., 2024). For instance, metabolic stress-induced mtDNA release in pancreatic β-cells activates cGAS–STING, leading to senescence-associated inflammation and impaired β-cell function, disrupting glucose homeostasis (Hu et al., 2022). More broadly, cGAS–STING upregulation under metabolic stress impairs insulin signaling and worsens glucose tolerance in obesity and non-alcoholic fatty liver disease models (Gong et al., 2024). In the nucleus, chromatin-bound cGAS recruits the SWI/SNF complex to drive glutaminolysis and metabolic gene expression programs (Lv et al., 2024).

Emerging studies further show that metabolic byproducts regulate cGAS activity. Lactate accumulation leads to lysine lactylation of cGAS, attenuating its immune function and shifting its role toward metabolic adaptation (Zhang et al., 2019a; Sun et al., 2023; Rao et al., 2025). This highlights a bidirectional regulatory loop: cGAS reshapes cellular metabolism while being modulated by the metabolic state itself.

Together, these findings underscore the central role of cGAS–STING in coordinating immune sensing with energy metabolism, positioning it as a critical node in immunometabolic crosstalk.

## Therapeutic implications: targeting cGAS in metabolic diseases

Given its central role in linking immune responses to metabolic regulation, cGAS has emerged as a compelling therapeutic target for metabolic disorders, including obesity, insulin resistance, MAFLD, and cardiometabolic diseases (Gong et al., 2024).

A range of therapeutic modalities have been explored to directly inhibit cGAS activity. Small-molecule inhibitors such as RU.521 and G140 have shown efficacy in blocking cGAS enzymatic activity *in vitro* and in preclinical models (Vincent et al., 2017; Lama et al., 2019). Antisense oligonucleotides offer gene-specific knockdown approaches (Valentin et al., 2021). VENT-03, a potent cGAS inhibitor currently in clinical trials for autoimmune diseases, such as systemic lupus erythematosus, underscores the translational potential of cGAS-targeted therapies (Mullard, 2023). Furthermore, studies in TREX1-deficient mice demonstrate that pharmacological cGAS inhibition can attenuate inflammation and extend survival (An et al., 2015; Dai et al., 2019; Liu et al., 2019; Tan et al., 2021). These findings collectively support the promise of cGAS inhibition in the context of immunometabolic diseases.

However, given the dual roles of cGAS in both immunity and metabolism, therapeutic interventions must strike a careful balance. Major challenges include achieving tissue-specific delivery (Ying et al., 2024; Shen et al., 2025), avoiding off-target effects, and accounting for compensatory activation of other immune pathways (Ying et al., 2024). Moving forward, research should prioritize the improvement of the delivery systems for targeted tissue-specific inhibition, the enhancement of specificity of therapeutic agents, and comprehensive monitoring of both metabolic and immune functions during treatment. Together, these strategies will be essential for safely and effectively harnessing cGAS inhibition in metabolic disease therapy.

To address these challenges, several strategies are emerging that aim to selectively modulate the metabolic functions of cGAS while preserving its immune surveillance capacity. One promising approach involves targeting STING-independent mechanisms, such as the cGAS–Beclin-1 interaction, which promotes mitophagy and mitochondrial quality control without triggering inflammatory cytokine production (Liang et al., 2014). Nuclear-localized ccGAS, which governs chromatin remodeling and metabolic gene expression independently of STING activation, is another attractive metabolic target (Lv et al., 2024).

Additionally, tissue-specific delivery of cGAS inhibitors—via nanoparticle encapsulation or ligand-directed targeting (Ying et al., 2024)—may confine their action to metabolically active tissues like the adipose tissue or liver, thereby sparing immune cells. Finally, recent studies highlight post-translational modifications, such as lactylation, which suppress cGAS immune activity while maintaining or altering its metabolic functions (Li et al., 2024). Isoform-specific activation of HIF-2α (e.g. via FG-4592) rebalances ceramide metabolism and alleviates insulin resistance while sparing inflammatory responses (Zhang et al., 2019b;

Li et al., 2021). Together, these precision strategies offer promising avenues for therapeutically harnessing cGAS in metabolic diseases without compromising host immunity.

**Figure 1:**
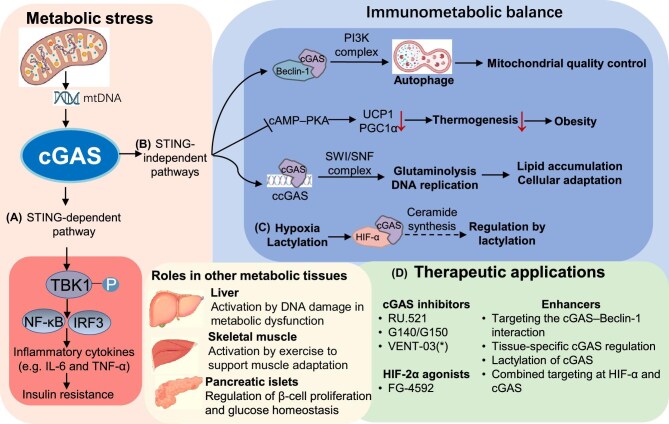
Schematic overview of cGAS-mediated immunometabolic regulation across metabolic organs and therapeutic strategies. Metabolic stressors such as high-fat diet and nutrient overload induce mitochondrial stress and mtDNA leakage in multiple organs, including the adipose tissue, liver, skeletal muscle, and pancreas, leading to cGAS activation. (**A**) STING-dependent pathway: mtDNA-activated cGAS triggers the STING–TBK1–NF-κB/IRF3 cascade, promoting the release of inflammatory cytokines (e.g. IL-6 and TNF-α) and impairing insulin signaling via the IRS–PI3K–AKT pathway. (**B**) STING-independent pathways: cGAS interacts with Beclin-1 to promote mitophagy, suppresses cAMP–PKA–UCP1/PGC1α cascade and thermogenesis, and modulates glutaminolysis via chromatin remodeling. (**C**) Hypoxia and lactylation: HIF-1α promotes ceramide synthesis and insulin resistance, while HIF-2α enhances ceramide degradation and alleviates insulin resistance; lactate-induced cGAS lactylation suppresses immune activation and shifts toward metabolic roles. (**D**) Therapeutic strategies include small-molecule inhibitors (e.g. RU.521, G140, and VENT-03), HIF-2α agonists (e.g. FG-4592), nanoparticle delivery systems, Beclin-1-targeted agents, and lactylation modulators. This figure illustrates how cGAS serves as a metabolic sentinel, integrating immune signaling and metabolic adaptation, and provides a visual guide to therapeutic opportunities.

A visual summary of these upstream activators, downstream branches, and therapeutic strategies is provided in Figure 1. This schematic integrates immunometabolic cGAS signaling in the adipose and other metabolic tissues, highlighting both STING-dependent inflammatory responses and STING-independent metabolic regulation pathways, as well as current and emerging intervention strategies.

## Future directions

Despite growing interest in the metabolic functions of cGAS, many important questions remain unanswered. While the adipose tissue has been a focus, the roles of cGAS in other metabolically active organs, such as the liver, skeletal muscle, kidney, heart, and pancreatic islets, are still not fully understood.

Another question is how cGAS operates independently of STING. Recent work has highlighted potential mechanisms involving cGAS interactions with nuclear DNA repair proteins, cytosolic metabolic enzymes, and autophagy regulators (Pan et al., 2023; Lu et al., 2024; Schmid et al., 2024). However, the detailed mechanisms remain largely unexplored. Advanced tools like proteomics and proximity labeling could help define the cGAS interactome across tissues and time.

From a translational perspective, it is crucial to target cGAS's metabolic roles without disrupting its immune defense functions. This requires precision strategies, such as small molecules or gene editing tools, which selectively act on the adipose or metabolic tissues. Tissue-specific knockout models, along with single-cell transcriptomics and lipidomics, will be vital to track these effects over time.

## Conclusion: a paradigm shift in cGAS biology

The role of cGAS, as both an immune sentinel and a metabolic regulator, in the adipose tissue exemplifies the intricate interplay between immunity and metabolism. Targeting cGAS offers a novel therapeutic avenue for treating metabolic disorders such as obesity, insulin resistance, and cardiometabolic diseases. However, developing such therapies requires a nuanced understanding of cGAS's multifaceted roles to ensure both efficacy and safety. A major challenge is to achieve metabolic benefits without compromising its essential function in innate immune defense.

To this end, a precision medicine approach is essential, which employs small molecules or gene-editing tools to selectively modulate cGAS's metabolic activities in the adipose tissue while preserving its immune functions in other cell types. To advance this selective targeting strategy, longitudinal and tissue-specific cGAS knockout models, coupled with single-cell transcriptomics and lipidomics, will be critical for mapping the tissue- and context-specific functions of cGAS throughout disease progression.

Ultimately, redefining cGAS as a dual-function molecule and central player in immunometabolism not only enhances our understanding of basic biology but also opens new frontiers for the development of next-generation therapies for metabolic diseases. By bridging current gaps between immunity and metabolism in metabolic disorders, we can fully harness cGAS in both metabolic research and clinical application.

## Supplementary Material

mjaf018_Supplemental_File

## Notes

*[This work was partially supported by grants from the Distinguished Young Scholar Foundation of Hunan Province (2023JJ10090) and the National Natural Science Foundation of China (82170886 and 82370858). J.W. drafted the manuscript. W.M. developed the initial concept and framework for the manuscript and revised the manuscript. Both authors contributed to the content, drafting, and critical review of the manuscript.]*
