# Supplementary material for: cGAS: bridging immunity and metabolic regulation
Source: J Mol Cell Biol. 2025 Jul 17;18:mjaf018. doi: 10.1093/jmcb/mjaf018 (PMC13344424; doi:10.1093/jmcb/mjaf018)
Supplement: mjaf018_Supplemental_File [file mjaf018_supplemental_file.docx]

Dear Editor,

We are pleased to submit our manuscript entitled "cGAS: Bridging Immunity and Metabolic Regulation" for consideration as a *Perspective* in *JMCB.*

Traditionally recognized as a cytosolic DNA sensor that activates innate immunity, cyclic GMP-AMP synthase (cGAS) has recently emerged as a critical regulator of cellular metabolism. In this Perspective, we propose a paradigm shift redefining cGAS as a dual-function molecule, an immune sentinel and a metabolic regulator. We provide a comprehensive overview of emerging evidence linking cGAS to adipose tissue dysfunction, insulin resistance, thermogenic suppression, and broader metabolic disturbances across multiple organs.

Importantly, we highlight the STING-independent metabolic functions of cGAS, including its roles in autophagy, mitochondrial integrity, chromatin remodeling, and hypoxia response. These findings position cGAS as a central node at the intersection of immunity and metabolism, with profound implications for diseases such as obesity, MAFLD, and cardiometabolic disorders.

We believe this Perspective is timely and significant, as it integrates recent discoveries from immunology, metabolism, and translational medicine, and outlines a forward-looking research agenda. As the field begins to explore cGAS-targeted therapies for autoimmune disease, we argue that selective modulation of cGAS may offer a new therapeutic avenue for metabolic disease, provided its immune functions are preserved.

This manuscript will be of broad interest to researchers in immunology, metabolism, endocrinology, and translational biology. We have no conflicts of interest to declare, and the manuscript has not been published or submitted elsewhere.

We sincerely thank you for considering our submission. We believe *JMCB* provides the ideal platform for sparking discussion and advancing the field’s understanding of cGAS in immunometabolism.

We would appreciate it if Drs. Hongting Zheng (Email: fnf7703@hotmail.com), Junli Liu (liujunli@fudan.edu.cn), Xian-Zheng [Zhang (xz-zhang@whu.edu.cn.),](mailto:Zhang(xz-zhang@whu.edu.cn.),) [Guo-Min Li](https://pubmed.ncbi.nlm.nih.gov/?term=Li+GM&cauthor_id=37640708) (Guo-Min.Li@UTSouthwestern.edu) who are experts in this area, could review our work.

Thank you very much for your consideration of our manuscript.

Sincerely,

Wen Meng, Ph.D.

Metabolic Syndrome Research Center

The Second Xiangya Hospital of Central South University

Changsha, Hunan, China
